# Development of a unilateral ureteral obstruction model in cynomolgus monkeys

**DOI:** 10.1002/ame2.12185

**Published:** 2021-11-19

**Authors:** Linghong Huang, Jia Ni, Tanika Duncan, Zhizhan Song, Timothy S. Johnson

**Affiliations:** ^1^ Immunology Therapeutic Area UCB Pharma Slough UK; ^2^ Research and Development Prisys Biotechnologies Pudong China; ^3^ Present address: Haisco Pharmaceutical Group Co., Ltd Chengdu China; ^4^ Present address: Experimental Renal Medicine, Oncology & Human Metabolism, School of Medicine University of Sheffield Sheffield UK

**Keywords:** animal models, fibrosis, kidney, nonhuman primates, transglutaminase

## Abstract

**Background:**

Chronic kidney disease (CKD) has a high global prevalence and large unmet need. Central to developing new CKD therapies are in vivo models in CKD. However, next‐generation antibody, protein, and gene therapies are highly specific, meaning some do not cross‐react with rodent targets. This complicates preclinical development, as established in vivo rodent models cannot be utilized unless tool therapeutics are also developed. Tool compounds can be difficult to develop and, if available, typically have different epitopes, sequences, and/or altered affinity, making it unclear how efficacious the lead therapeutic may be, or what dosing regimen to investigate. To address this, we aimed to develop a nonhuman primate model of CKD.

**Methods:**

In vivo rodent unilateral ureteral obstruction (UUO) models kidney fibrosis and is commonly used due to its rapidity, consistency, and ease. We describe translation of this model to the cynomolgus monkey, specifically optimizing the model duration to allow adequate time for assessment of novel therapeutics prior to the fibrotic plateau.

**Results:**

We demonstrated that disease developed more slowly in cynomolgus monkeys than in rodents post‐UUO, with advanced fibrosis developing by 6 weeks. The tubulointerstitial fibrosis in cynomolgus monkeys was more consistent with human obstructive disease than in rodents, having a more aggressive tubular basement expansion and a higher fibroblast infiltration. The fibrosis was also associated with increased transglutaminase activity, consistent with that seen in patients with CKD.

**Conclusion:**

This cynomolgus monkey UUO model can be used to test potential human‐specific therapeutics in kidney fibrosis.

## Graphical abstract

Next‐generation antibody, protein, and gene therapies are highly specific, meaning some do not cross‐react with rodent targets. We developed a nonhuman primate model of chronic kidney disease by translating rodent unilateral ureteral obstruction models to cynomolgus monkeys. This nonhuman primate model developed advanced fibrosis by 6 weeks that was more consistent with human pathology than rodent models and can be applied to evaluate the potential of human‐specific therapeutics in kidney fibrosis.

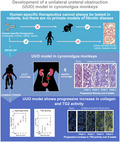

## 1 INTRODUCTION

Chronic kidney disease (CKD) is one of the most common diseases worldwide with kidney fibrosis being a histological hallmark.^1^, ^2^ Several animal models of CKD have been developed to understand pathogenesis and verify disease targets for therapeutic treatment. Unilateral ureteral obstruction (UUO) is a model resembling human obstructive nephropathy. Although it is not a common cause of human renal disease, it is a well‐recognized and frequently used model of kidney damage with subsequent fibrosis development.^3^, ^4^, ^5^ By ligating a ureter of one kidney, the build‐up of urine elevates intratubular pressure; this results in reduction in renal blood flow and glomerular filtration rate, as well as other changes such as interstitial inflammation, tubular dilation, tubular atrophy, and ultimately, fibrosis. Rodent UUO models have been utilized to investigate mechanisms of tubulointerstitial fibrosis, with severe fibrosis being detected within 21 days.^6^, ^7^

Development of fibrosis in the rodent UUO model is associated with changes in extracellular transglutaminase (TG) 2^8^ and is a hallmark of human disease pathology.^9^ TG2 is a calcium‐dependent enzyme that crosslinks extracellular matrix (ECM) proteins to form a stable and proteolytic resistant ε(γ‐glutamyl)‐lysine dipeptide crosslink.^10^ This increased crosslinking in ECM accelerates the rate of ECM deposition, while making it less susceptible to degradation by ECM proteases. Increases in extracellular TG2 expression, TG activity, and ε(γ‐glutamyl)‐lysine dipeptide crosslinks have been demonstrated in all types of human CKD, and are highly correlated with fibrosis levels.^9^ Studies performed using rodent models of kidney fibrosis have shown increased TG2 and that blocking its activity slows renal fibrosis.^8^, ^11^, ^12^, ^13^ A study is currently underway to evaluate inhibition of TG2 in patients with CKD (NCT04335578). TG2 is therefore a useful mechanistic tool to evaluate if the pathological processes of CKD occurring in an in vivo model are consistent with those in humans.

Although some proteins are homologous between species, antibodies targeting functional human epitopes (i.e., therapeutic antibodies) can be highly species‐specific as they often recognize conformational epitopes, whereby changes in a single amino acid in the epitope can raise affinity and lower half‐maximal inhibitory concentration (IC_50_) significantly. Therefore, antibodies optimized for human proteins may not sufficiently cross‐react with other species to allow testing in commonly used rodent in vivo models. An option to mitigate this is to use a surrogate tool antibody; however, binding at a different epitope may complicate interpretation of data, provide different pharmacokinetics and thus, less valuable translational data to predict efficacious doses in early clinical studies. It is becoming increasingly important to use models in species with high homology and close physiology to humans to evaluate next‐generation therapeutic antibodies and other modalities where species homology plays a significant role such as gene, small interfering RNA (siRNA), and oligonucleotide therapies. The nonhuman primates (NHP) most commonly used in medical research are from the genus *Macaca*, or more specifically the cynomolgus monkey. Their consensus with the human proteome^14^ allows a high degree of cross‐reactivity, making them a suitable species for studies with highly specific antibodies or other new therapeutic modalities. Models of liver^15^ and lung fibrosis^16^ have been established in cynomolgus monkeys and there are NHP models of renal transplantation/rejection.^17^ However, to date, there are no models of renal fibrosis in NHP. Therefore, we hypothesized that a cynomolgus monkey UUO model could be developed and validated. The UUO model surgery is simple (compared with other models such as 5/6th subtotal nephrectomy) and the remaining functional kidney prevents problems with organ failure and any associated welfare issues such as end‐stage renal failure. Translating the UUO model from mice to rats to rabbits was relatively consistent and quick; thus, we did not anticipate issues in translating the model to cynomolgus monkeys. Given the biggest variable between species when using the UUO model is the time to develop fibrosis consistent with end‐stage renal disease, we undertook a time‐course evaluation of tubulointerstitial fibrosis post‐UUO to understand the optimal duration of the model and evaluate expression of a human‐relevant pathological process currently being tested in the clinic: TG2.

## 2 MATERIALS AND METHODS

### 2.1 Animal study

Fourteen male cynomolgus monkeys (Hainan Jingang Biotech Co. Ltd., China) were used in the study. All animals were tuberculosis‐negative and free of viral, bacterial, or parasitic infections (including simian immunodeficiency virus, respiratory syncytial virus, simian‐T‐lymphotropic virus, tuberculosis, Schmallenberg virus, shigellosis, and salmonella). Adult monkeys (aged 7–8 years) were used in this study to more accurately model human disease. Common tests for kidney disease were performed to ensure that the animals had no proteinuria (urinary protein <10 mg/dl, test with visual urinary test strip) or loss of renal function (serum creatinine <100 µmol/L, blood urea nitrogen <9 mmol/L) at study initiation.

Each monkey was housed in a stainless steel cage with a minimum living area of length 160 cm × depth 80 cm × height 190 cm at 18–29°C with a relative humidity of 40%–70% and had access to the exercise cage sized 480 × 80 × 190 cm for 24 h each week (1 animal per cage). Monkeys had free access to monkey chow (Guangzhou Guolong Science & Technology Co. Ltd., China) and municipal tap water meeting drinking standards; vegetables and fruit treats were also given daily to stimulate their appetite.

The 14 cynomolgus monkeys were randomized (simple randomization method) into 5 groups: 1 consisting of 2 sham‐operated animals (harvested at week 6; one time point was sufficient for the sham population, to confirm that no fibrosis occurred at the maximum time point) and 4 groups of 3 animals undergoing UUO and kept for experimental periods of 3, 4, 5, or 6 weeks after surgery. Sham animals received laparotomy only and animals in the UUO groups had the left ureter occluded with a double silk tie. Animals were fasted for 12 h and water withheld for 3 h prior to general anesthesia. Anesthesia was induced by intramuscular (IM) injection of 5 mg/kg Zoletil 50 (Virbac, Carros, France) initially and maintained using 5% isoflurane inhalation. Prior to a midline incision in the abdominal wall, fur was shaved and skin disinfected. The ureter of the left kidney was dissected and ligated at 2 adjacent points by silk ties and the abdominal wall subsequently closed. To minimize infection, 25 000 IU/kg of penicillin was given by IM injection twice daily for 3 days. The analgesia regimen applied is listed in Table 1. Animals were closely monitored post‐surgery; their appearance, body weight, temperature, respiratory rate, and behavior were assessed daily and their skin wound checked by an onsite vet every day until it healed. All UUO animals were provided with soft bedding after surgery until normal behavior resumed (scored and recorded during each observation). In general, the UUO surgical procedure was associated with moderate pain, and all animals recovered within a few days. At harvest, animals were euthanized by intravenous injection of 80 mg/kg pentobarbital, and kidneys dissected longitudinally and each half cut into 4 segments. One segment from each half kidney was fixed in 10% neutral formalin solution and embedded in paraffin blocks. The other segments were snap frozen and used for in situ TG activity, extracellular TG2 staining, and hydroxyproline detection. Researchers performing sample analysis did not know the group allocation until data were generated.

**TABLE 1:** Analgesia regimen

Drug	Dose	Dosing time	Frequency	Duration
Codeine	0.5 mg/kg, PO	2 h before surgery	3 times daily	3 days
Lornoxicam	0.7 mg/kg, IM	20 min before surgery	Once daily	—
Lornoxicam	0.35 mg/kg, IM	11 h post‐surgery	Twice daily	3 days

Abbreviations: IM, intramuscular; PO, oral administration.

### 2.2 Picrosirius red and Masson's trichrome stain

Neutral‐buffered, formalin‐fixed, paraffin‐embedded 4 µm sections were dewaxed and rehydrated. Nuclei were stained with Weigert's hematoxylin (Sigma Aldrich, UK), washed in water, and stained in Picrosirius red (PSR) solution for 1 h prior to being washed in acidified water. Sections were dehydrated and cleaned using a series of alcohols and xylenes and then mounted in dibutylphthalate polystyrene xylene (DPX). Masson's trichrome stain was then performed using a kit (Sigma Aldrich, UK) as per manufacturer instructions (stains collagenous material blue, nuclei brown and fibers, erythrocytes and elastin red/pink).

### 2.3 In situ TG activity

Detection of in situ TG activity was performed as described previously^13^ with some modification. Frozen kidney tissues were mounted in optimal cutting temperature (OCT) mounting media (VWR International, UK) and cut at 10 µm thickness. Unfixed sections were washed in phosphate‐buffered saline (PBS) and incubated with reconstitution PBS made up of 5% bovine serum albumin (BSA) with protease inhibitors (Sigma Aldrich, UK) for 30 min at room temperature. Sections were then washed twice with PBS prior to being incubated with reaction mix containing 5 mmol/L CaCl_2_, 1 mmol/L dithiothreitol, and 0.5 mmol/L biotin cadaverine (Thermo Fisher Scientific, UK) with protease inhibitors in 50 mmol/L Tris (pH 7.4) for 30 min at 37°C. A negative control in which CaCl_2_ was replaced with 10 mmol/L ethylenediaminetetraacetic acid was used. Slides were washed 3 times with PBS, fixed in cold acetone for 5 min, and air dried. Sections were washed in PBS and incubated with a 1 in 500 dilution of streptavidin‐Alexa 555 (Thermo Fisher Scientific, UK) for 1 h at 37°C. Slides were then washed 3 times with PBS and mounted with anti‐fade mounting media containing 4′,6‐diamidino‐2‐phenylindole (DAPI).

### 2.4 Extracellular TG2 antigen staining

Immunodetection of extracellular TG2 was performed as previously described.^9^, ^12^ Frozen kidney tissues were mounted in OCT mounting media and cut at 10 µm thickness. Unfixed sections were washed in PBS, and blocked in PBS buffer made up of 5% BSA with protease inhibitors for 30 min at room temperature prior to being incubated with a mouse anti‐human TG2 antibody (IA12; generated recombinantly in‐house at UCB, using the sequences described in patent WO/2013/175229^18^ and used previously for analytical work^8^) at a concentration of 30 µg/mL for 1 h at 37°C. Sections were washed 3 times with PBS, fixed in cold acetone for 5 min, and air dried. These were then washed with PBS before a goat anti‐mouse Alexa 488 antibody (Thermo Fisher Scientific, UK), diluted 1 in 500 in 5% BSA/PBS, was added. After incubation for 1 h at 37°C, sections were washed in PBS and mounted with anti‐fade mounting medium containing DAPI.

### 2.5 Measurement of kidney fibrosis levels, in situ TG activity and extracellular TG2 protein

**FIGURE 1:**
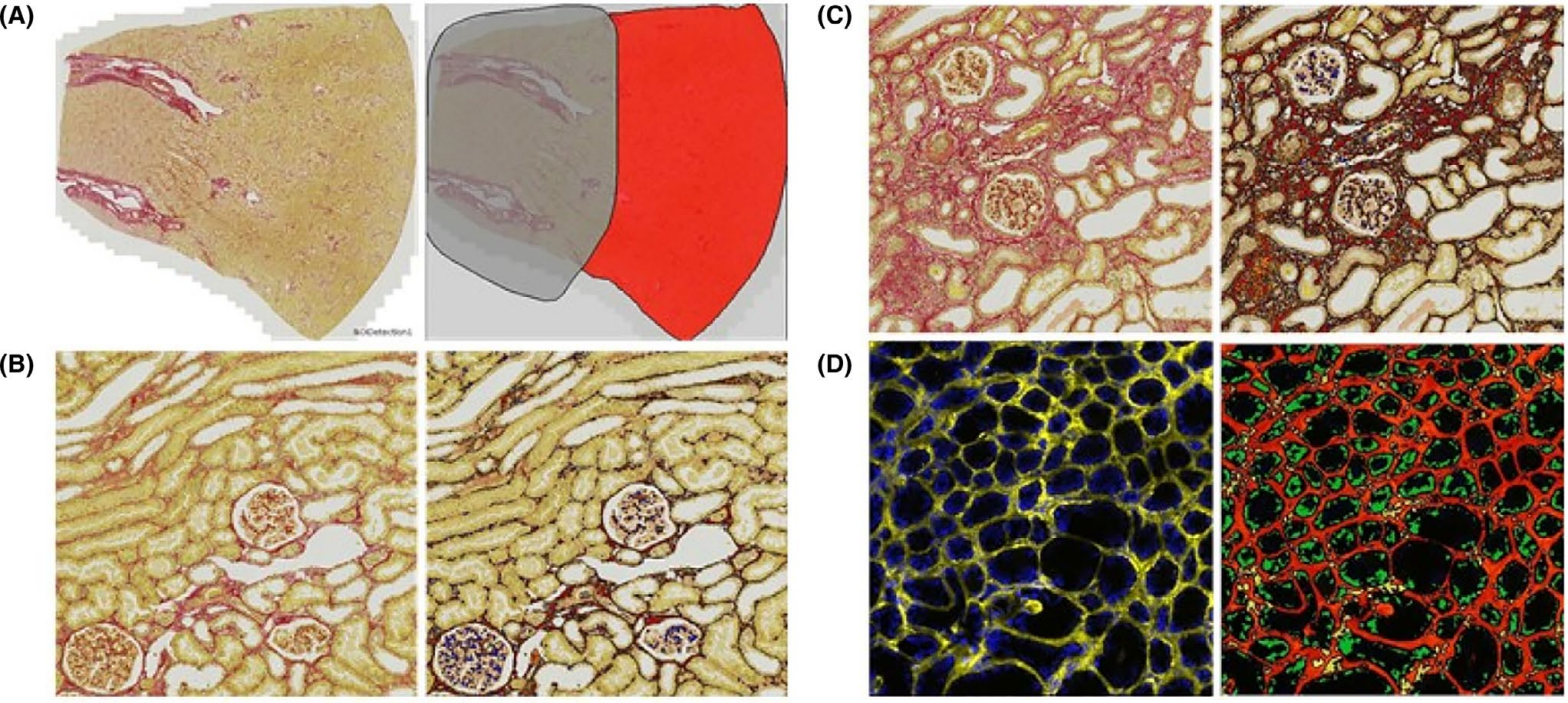
Example of ROI selection (A), cellular analysis on normal tissue (B) and fibrotic tissue (C), and analysis on a TG activity image (D). ROI selection (A) is demonstrated by the autodetected tissue area (red) and the manually excluded medulla area (gray). Red collagen stain is labeled in red with intense staining overlaid in orange by cellular analysis. Nuclei are marked in blue (B, C). TG activity shown in yellow is covered by red and blue nuclei overlaid in green (D). ROI, region of interest; TG, transglutaminase

Images of PSR‐stained sections were downloaded onto Tissue Studio (Definiens Inc., USA). Selection of the region of interest (ROI) was performed on sections, separating out the cortex for analysis (Figure 1A). Definiens Stain Picker options and “computer training/learning” methods were used to generate an algorithm to identify collagen staining (red and intense staining overlaid in orange) and nuclei (blue) (Figure 1B,C). Definiens automatically calculates the areas of the ROI, and the 3 marker areas for each slide. The ratio of collagen area to cortical area was calculated to represent the fibrotic index.

To analyze images of TG activity and extracellular TG2 protein, the dedicated Definiens Fluorescence workspace was used. ROI detection was performed automatically and corrected manually to remove the medulla from analysis. Marker detection was then performed on different image layers depending on the marker. DAPI nuclear stain (blue) was projected as green and analyzed on layer 1 (green overlay), and TG activity (yellow) or extracellular TG2 (green) was projected as red and analyzed on layer 2 (red overlay, intense staining overlaid in yellow) (Figure 1D). The area of staining was determined by the Definiens software, and levels of TG activity or extracellular TG2 were calculated using the ratio of the staining area to DAPI nuclear stain area.

### 2.6 Hydroxyproline analysis

Kidney homogenates containing 5 mg of protein per sample were hydrolyzed in 6 mol/L hydrochloric acid at 110°C for 24 h. These were centrifuged at 18 000 g for 2 min and the supernatant transferred to clean tubes and freeze dried. Samples were resuspended in 500 µl of lithium loading buffer (Biochrom, UK) and 30 µl fractionated using a lithium chloride gradient on a Biochrom 30+ amino acid analyzer using the manufacturer's standard protocol and expressed as µmol/mg protein.

### 2.7 Measurement of ε‐(γ‐glutamyl)‐lysine crosslink

Kidney homogenate containing 10 mg of protein from each animal was subjected to exhaustive proteolytic digestion with subtilisin, pronase, leucine amino peptidase, prolidase, and carboxypeptidase Y. Measurement of ε‐(γ‐glutamyl)‐lysine crosslinks was performed using cation exchange chromatography on an amino acid analyzer (Biochrom, UK).

### 2.8 Statistical analysis

Raw data are shown with each symbol representing one animal. Data analyses were performed using one‐way analysis of variance (ANOVA) by a Fisher's least significant difference multiple comparisons test (GraphPad Prism). A significance level of 5% (*p* < .05) was adopted throughout.

## 3 RESULTS

### 3.1 Kidney fibrosis

**FIGURE 2:**
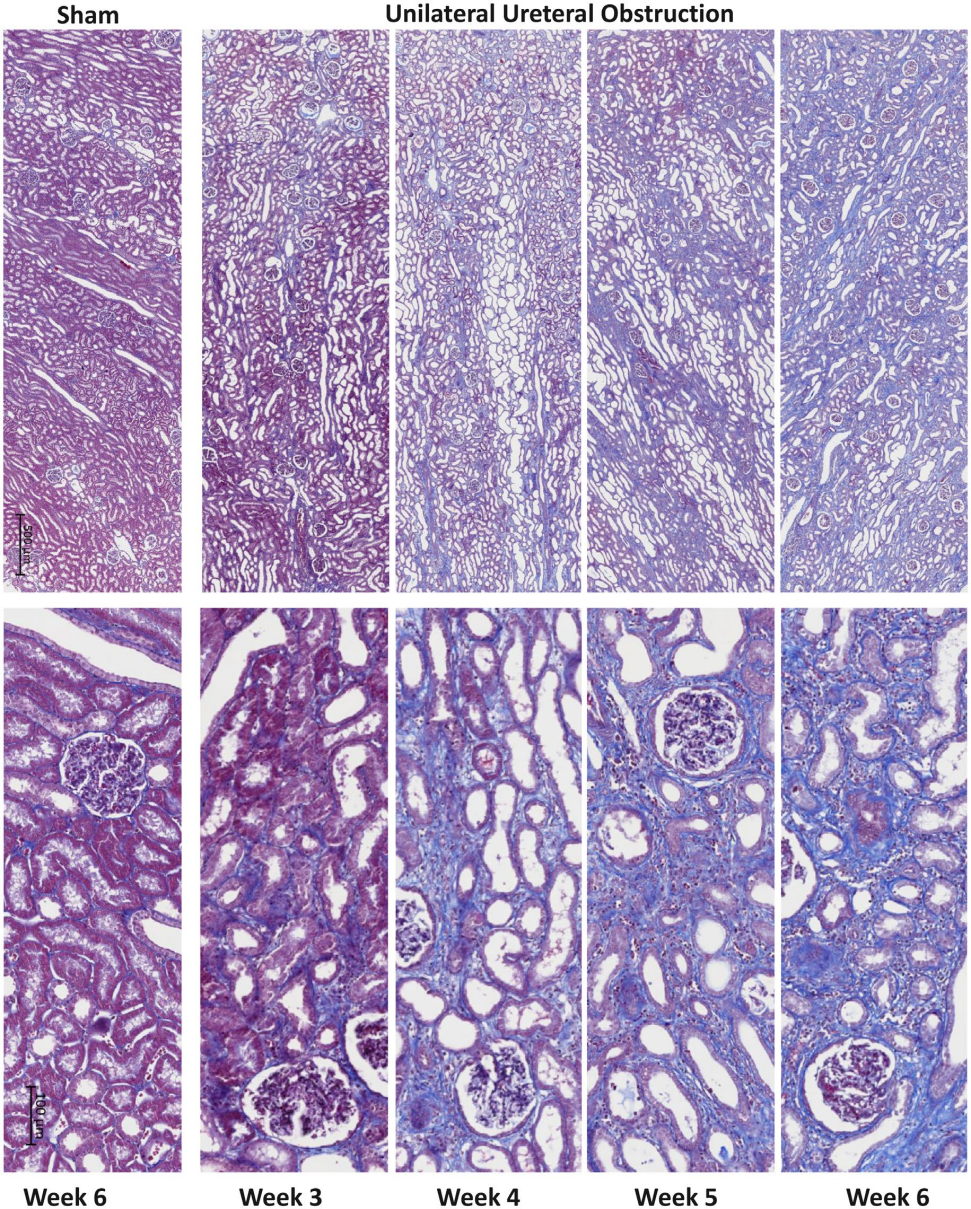
Progression of kidney fibrosis post‐UUO, with Masson's trichrome staining. Kidney segments were fixed in 10% neutral formalin solution for 24 h and blocked. Paraffin embedded 4 µm tissue sections were stained for Masson's trichrome. Collagen is stained blue, nuclei brown, and fiber, erythrocytes, and elastin red/pink. UUO, unilateral ureteral obstruction

**FIGURE 3:**
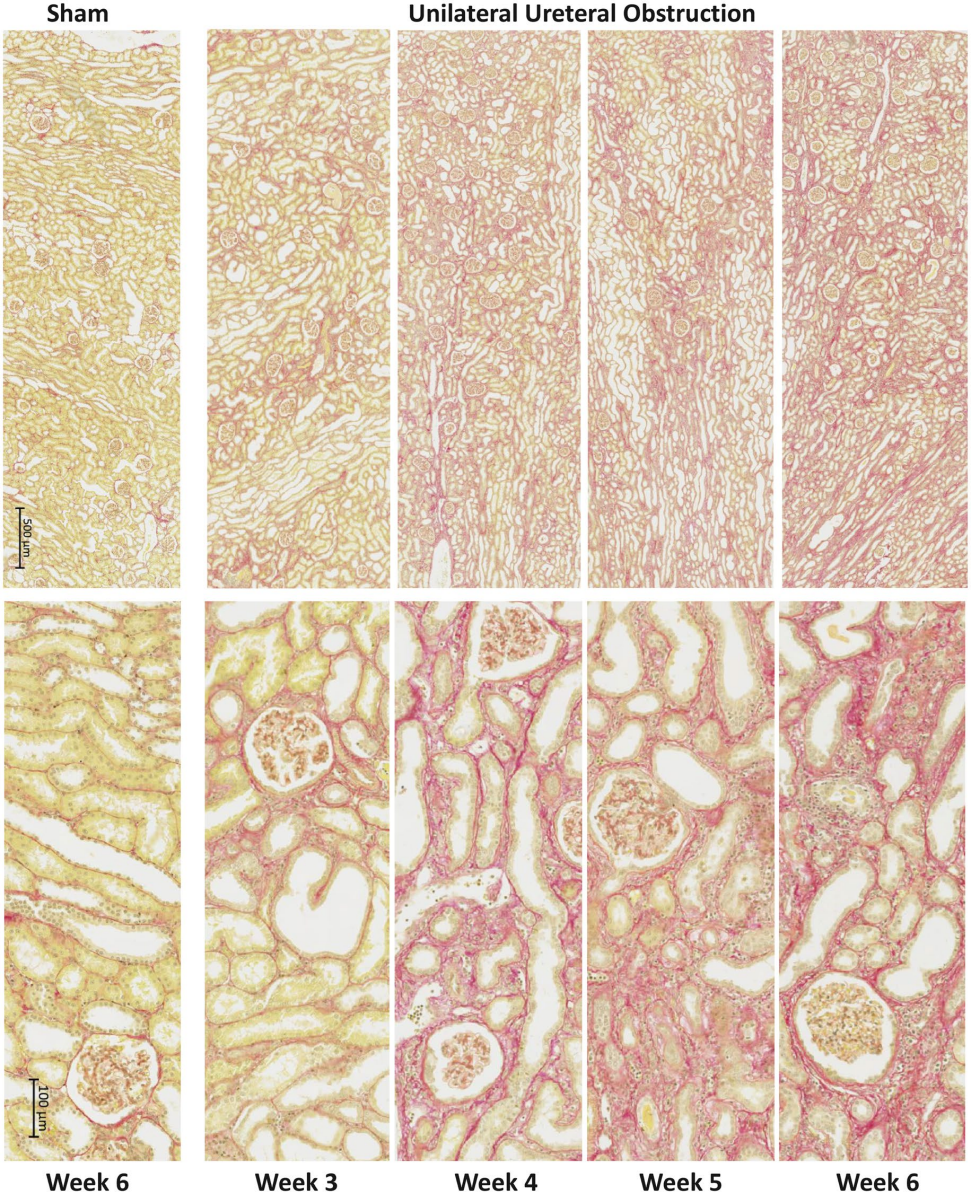
Progression of kidney fibrosis post‐UUO, with PSR staining. Kidney segments were fixed in 10% neutral formalin solution for 24 h and blocked. Paraffin embedded 4 µm tissue sections were stained with PSR. Collagen is stained in red and cytoplasm in yellow. PSR, picrosirius red; UUO, unilateral ureteral obstruction

**FIGURE 4:**
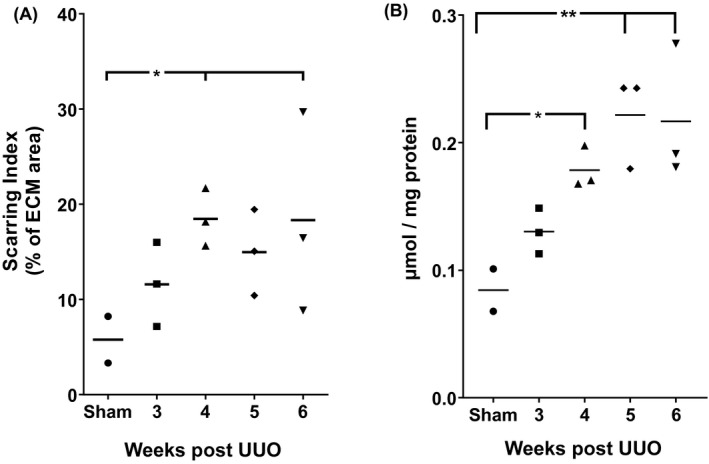
Measurement of kidney fibrosis in cynomolgus monkey UUO model. Images of PSR‐stained sections were analyzed using Definiens Tissue Studio software. Cortical area and area covered by red in the kidney cortex were quantified. (A) PSR staining. Fibrosis levels are expressed as the percentage of red (collagen) in the cortical areas. (B) Kidney hydroxyproline. Levels of hydroxyproline are expressed as µmol per mg protein. Raw data are presented with the line indicating the mean. * and ** indicate *p* < .05 and *p* < .01, respectively (one‐way ANOVA). ECM, extracellular matrix; PSR, picrosirius red; UUO, unilateral ureteral obstruction

At 6 weeks, UUO in cynomolgus monkeys caused changes mainly in the tubulointerstitium, as expected. From week 3 onwards, under low magnification (Figure 2, upper panel), key changes observed were tubular dilation and increased collagen accumulation, shown as blue on the Masson's trichrome stained images. Using higher‐power magnification (Figure 2, lower panel), it was possible to observe flattened and elongated tubular epithelial cells leading to dilated tubules with loss of the brush border. Thickening of the tubular basement membrane was particularly clear and notably in excess of that seen in rodent UUO models, as well as increased cell numbers in the expanded tubulointerstitial space. Compared with the sham, these changes were relatively mild in the 3‐week group, but progressed in severity through weeks 4 and 5, peaking at week 6. Of note, there was significant expansion of the tubular basement between weeks 3 and 4, which seemed to expand little after week 4 where tubular destruction and atrophy became the main histological changes. Fibrotic changes, especially tubulointerstitial expansion, was confirmed by PSR‐stained sections (Figure 3). Image analysis was performed to quantify levels of fibrosis. All UUO groups had increased fibrosis, reaching significance in the 4‐ and 6‐week UUO groups (Figure 4A).

Hydroxyproline is a major component of collagen and can be used as an easily quantifiable surrogate of the total amount of collagen present in the kidney. In this cynomolgus monkey model, significantly elevated hydroxyproline was detected in the UUO kidneys from week 4 onwards (Figure 4B). Compared with the sham, levels of hydroxyproline were increased by 1.5‐fold at week 3, more than double at week 4 and peaked with a 2.6‐fold change at weeks 5 and 6.

### 3.2 In situ TG activity, extracellular TG2 expression and levels of ε‐(γ‐glutamyl)‐lysine crosslink

**FIGURE 5:**
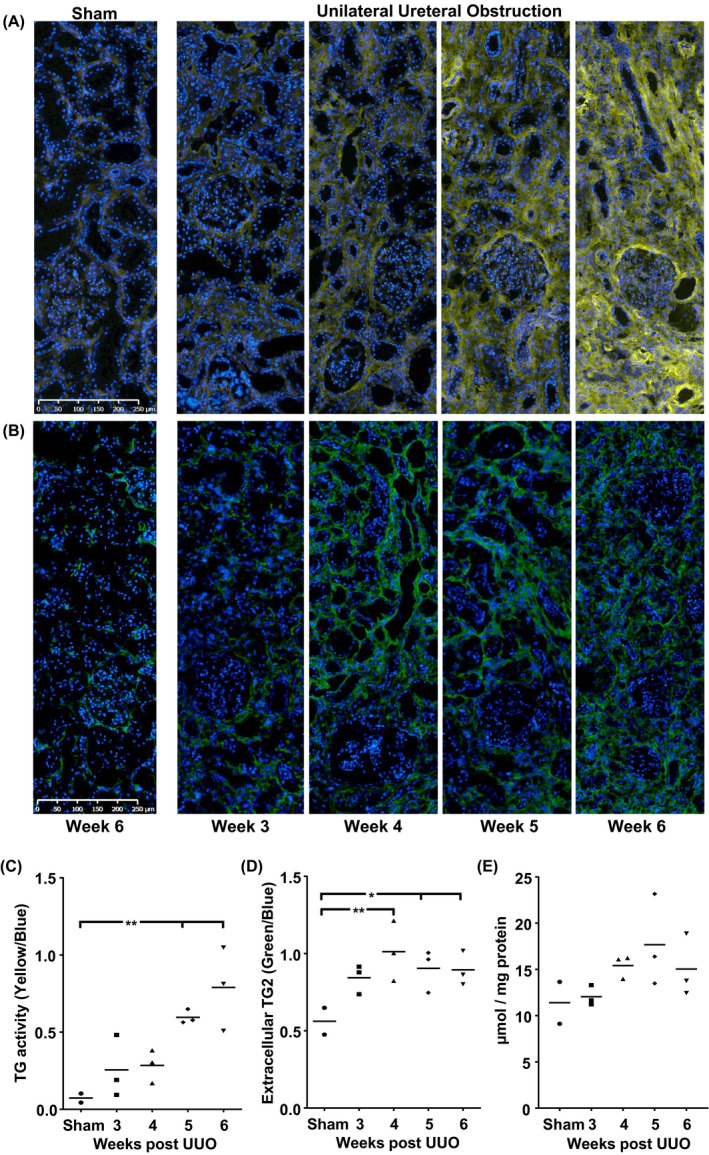
Changes in TG2 in cynomolgus monkey UUO kidneys. In situ TG activity (A) was detected on unfixed kidney cryostat sections by the incorporation of biotin cadaverine, which was revealed using a streptavidin‐Alexa 555 conjugate. TG activity is represented by yellow and nuclei stained as blue. Extracellular TG2 (B) was detected on unfixed kidney cryostat sections. TG2 antigen is represented by green and nuclei stained as blue. In situ TG activity (C) and extracellular TG2 (D) in the kidney cortex was quantified using Definiens Tissue Studio software. Levels of activity and TG2 expression were calculated by measuring the area of yellow and green staining. Values were corrected for cell number by calculating this as a fraction of the blue staining of nuclei. ε‐(γ‐glutamyl)‐lysine crosslink (E) was measured by releasing the dipeptide from 10 mg protein using exhaustive proteolytic digestion followed by cation exchange chromatography on an amino acid analyzer (Biochrom). Raw data are presented with the line indicating the mean. * and ** indicate *p* < .05 and *p* < .01, respectively (one‐way ANOVA). TG, transglutaminase; UUO, unilateral ureteral obstruction

In the healthy (sham) kidney, low TG activity was seen in the glomeruli and tubular basement membrane/tubulointerstitial space, based on incorporation of the labeled TG substrate (biotin cadaverine) (Figure 5A). Activity increased 3 weeks post‐UUO, with an elevation in substrate incorporation in the expanded tubulointerstitial space at 4 weeks post‐UUO. TG activity at 5 and 6 weeks post‐UUO continued to increase (Figure 5A) in contrast to TG2 extracellular antigen, which plateaued at weeks 5 and 6 (Figure 5B). When image analysis was performed to quantify changes in TG activity, 3‐, 6‐, and 8‐fold increases were seen at weeks 4, 5, and 6, respectively (Figure 5C).

In the kidneys of healthy (sham) cynomolgus monkeys, small amounts of extracellular TG2 were detected and located in the tubular basement membrane/tubulointerstitial space, periglomerular area and mesangial matrix (Figure 5B). In the kidneys of cynomolgus monkeys with UUO, changes in extracellular TG2 expression were mainly focused around tubules and associated with the tubular basement membrane (Figure 5B). Elevation in extracellular TG2 antigen reached a maximum at 4 weeks post‐UUO and remained at that level for the remainder of the study. Quantification of the image analysis indicated that extracellular TG2 increased in the kidneys of cynomolgus monkeys with UUO from 3 weeks onwards, with significant changes compared with the sham group at 4, 5, and 6 weeks post‐UUO (Figure 5D).

Analysis of ε‐(γ‐glutamyl)‐lysine crosslink in kidneys with UUO showed a progressive trend of elevated crosslink dipeptide that reached a maximum at 5 weeks post‐UUO and remained high to week 6 (Figure 5E).

### 3.3 Correlations between levels of in situ TG activity versus ε‐(γ‐glutamyl)‐lysine crosslink and hydroxyproline

**FIGURE 6:**
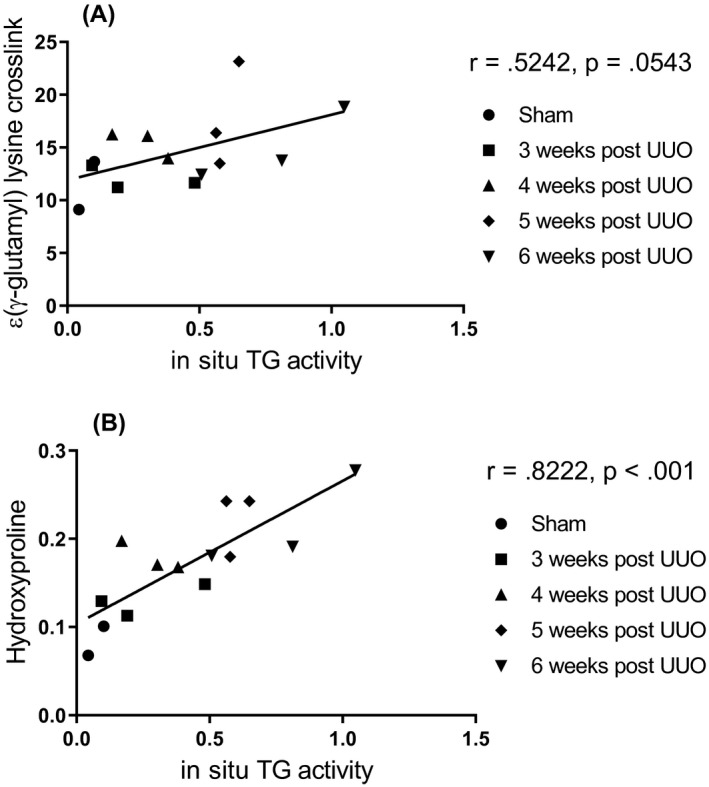
Correlations between in situ TG activity and ε‐(γ‐glutamyl)‐lysine crosslink (A), and between in situ TG activity and hydroxyproline (B). Mean of ε‐(γ‐glutamyl)‐lysine crosslink, in situ TG activity and hydroxyproline for each animal were calculated and used for correlation analysis, which was performed using a Pearson's correlation analysis. TG, transglutaminase; UUO, unilateral ureteral obstruction

Correlation between in situ TG activity and ε‐(γ‐glutamyl)‐lysine crosslink was *r* = .52 (Figure 6A). There was also a correlation between in situ TG activity and hydroxyproline, strongly linking the accumulation of collagen with TG2 in this model (*r* = .82, Figure 6B).

## 4 DISCUSSION

This study sought to transfer the UUO model of CKD from rodents to cynomolgus monkeys, with the aim of allowing development of human‐specific therapeutics, not cross‐reactive with rodents and with a novel mode of action, for which in vivo pharmacological data are essential to define dosing for phase I human studies. Understanding the time course of the disease process was key to allowing optimal selection of study duration and relating this to changes in collagen levels (and thus fibrosis), while using TG2 as an exemplar molecule of clinical stage target in CKD. The model was developed in adult animals to assist with translation, as CKD is typically a disease of later life in which changes occur in multiple parameters associated with fibrosis, including cell senescence,^19^, ^20^ promoter methylation,^21^ ECM homeostasis,^22^ and hemodynamics.^23^

Overall, these data demonstrate a clear development of tubulointerstitial fibrosis over the 3‐ to 6‐week study period, although data from the different methods used to assess the extent of fibrosis in this study were not totally aligned. Manual assessment of the sections would suggest fibrosis progressed over the 6‐week study period, approaching end‐stage remodeling around or just after week 6. Image analysis of PSR suggested that the level of interstitial collagens plateau from week 4 onwards, whereas total kidney hydroxyproline is progressive until week 5. In terms of TG2, the level of activity progressed over the 6 weeks, with maximum extracellular TG2 antigen reached by week 4, and the formation of ε‐(γ‐glutamyl)‐lysine peaking at week 5. This poses the question of the optimal time to run the model when being used to test interventional efficacy. It is generally accepted that the level of fibrosis will ultimately reach a plateau in non‐functional models such as the UUO. Therefore, model duration must be optimized to allow the maximum window for measurement of interventional success before the linear formation of scar tissue declines and hides any beneficial effect on slowing progression of fibrosis. Based on the composite data here, a 4‐week model in aged cynomolgus monkeys would provide the optimal balance between the disease window and the slowing of histological and mechanistic changes that underpin fibrosis. However, given the heterogeneity of data due to the intentionally small animal numbers used, it is possible that the optimal duration of this model to maximize the disease window could be 5 weeks.

Although the data allow us to conclude successful model development, there are 3 reasons for the variability observed in some of our data. Firstly, there are ethical considerations associated with any study in primates. It is important to use the smallest number of animals possible; however, it was difficult to calculate the absolute minimum number required as there is no precedent or data on which to base power calculations. Furthermore, establishing which time points to investigate proved challenging, given the limited data available. After reviewing early unpublished pilot work performed by Prisys Biotechnologies on 4 animals, it was decided that 3 animals at 4 time points would be used. This small number of animals used makes it hard to draw definitive conclusions on the data due to the variation in their responses. Secondly, it was our aim to develop a model in adult animals (7–8 years old) to more accurately model human disease. However, adult animals are more likely to have preexisting diseases; as such, all animals were prescreened for serum creatinine, blood urea nitrogen, and urine protein using a test strip, and only animals with results within a normal range studied. However, in some of the sections from sham animals, there were still some lesions with tubular dilation that exceeded that expected of 7‐ to 8‐year‐old monkeys. If these were also present in some of the animals receiving UUO, the rate and level of disease post‐UUO may have been affected. In hindsight, the urine test strip‐based screening process had insufficient sensitivity and resolution to detect existing kidney damage. To better standardize the baseline clinical parameters of the kidney, more sophisticated quantitative assays to measure levels of proteinuria and kidney function should be employed to allow selection of more homogeneous groups. In subsequent application of this model, we quantified proteinuria and protein and albumin‐to‐creatinine ratio, in samples collected 2 weeks apart; this led to exclusion of >40% of animals screened but significantly reduced data variability. Thirdly, Prisys Biotechnologies has access to study facilities on the same site as the breeding facilities, removing the need for animal transport and lowering animal stress and anxiety as their environment is unchanged. While this best practice approach undoubtedly lowers variability in disease stimuli, unlike laboratory rodents, cynomolgus monkeys are outbred. Therefore, despite reducing variability where possible, breeding from parents not closely related introduces genetic variation, adding significant heterogeneity to the system.

Despite variability in the data, we were able to make some key observations about the effect of UUO of the kidneys. Fibrotic changes in the kidney cortex shown by image analysis of PSR staining were consistent with levels of fibrosis, reaching a plateau from 4 weeks post‐UUO. This is slightly different to the hydroxyproline data that showed fibrosis reached plateau at 5 weeks post‐UUO, while visual histopathological assessment was progressive over the 6‐week period. The difference between collagen measures may be due to medullary tissue being included when hydroxyproline was measured but excluded in the PSR analysis in stained sections. Visually, the expansion of the tubular basement membrane reached a maximum at around 4 weeks, whereas disease progression is more often associated with subsequent tubular atrophy. As the tubular basement membrane area would be the primary parameter in high content image analysis of PSR, it is not surprising to note small increases in staining post 4 weeks. In addition, changes in TG activity do not seem to chronologically match levels of extracellular TG2 in the UUO kidney, with progressive TG activity over 6 weeks and TG2 antigen plateauing from 4 weeks post‐UUO. However, TG2 in the extracellular space may not be active, with studies showing rapid deactivation of extracellular TG2^24^ or TG2 being kept in its inactive state by binding to heparan sulphate proteoglycans such as syndecan 4.^25^ Thus, it is possible that there was a higher percentage of active TG2 in the UUO kidney at the later stages. Importantly, levels of in situ TG activity were positively correlated with changes in collagen quantity.

In conclusion, this study has successfully transitioned the rodent UUO model of CKD to a NHP. The model progresses to completion in approximately 6 weeks compared with 3 weeks in mice and rat models, with a suggestion that an optimal study period for testing therapeutics to avoid disease plateauing would be 4 weeks. The NHP model demonstrates a much more aggressive tubular interstitial fibrosis than rodents, with a larger expansion of the tubular basement membrane and greater infiltration of interstitial cells giving a histological picture closer to obstructive disease in humans. This NHP model can be applied to test human‐specific anti‐fibrotic therapeutic molecules to determine efficacy, target engagement, and pharmacokinetics.

## CONFLICT OF INTEREST

L.H., T.D., and T.S.J. are employees of UCB Pharma and may hold/have access to stock options. Z.S. is an employee of Prisys Biotechnologies Co., Ltd., and J.N. was employed by Prisys Biotechnologies Co., Ltd. at the time this work was conducted.

## AUTHOR CONTRIBUTIONS

All authors reviewed the manuscript and approved the final version for submission. L.H. was the lead scientist and wrote the manuscript. She developed, optimized, and ran the various analyses used as well as collating and analyzing the data generated. J.N. helped design the study and ran the in‐life phase of the investigation. T.D. performed Masson's trichrome and Picrosirius staining. Z.S. directed the animal study at Prisys Biotechnologies. T.J. designed the study, commissioned the work, and edited the manuscript.

## STATEMENT OF ETHICS

Animal work was approved by Prisys Institutional Animal Care and Use Committee under study number 2015‐PS11‐002 (license code SYXK‐2014‐007). UCB representatives (including the company‐named animal care and welfare officer) assessed facilities and procedures at Prisys Biotechnologies before commencement of studies to ensure that all were compatible with UK Home Office guidelines, for the use of experimental animals as defined under the Animal Scientific Procedures Act 1986 (ASPL), and that current ‘best practice’ in animal welfare was being applied. A UCB representative was subsequently present during all experimental procedures to ensure full compliance with ASPL.
